# The role of β-hydroxybutyrate testing in ketogenic metabolic therapies

**DOI:** 10.3389/fnut.2025.1629921

**Published:** 2025-09-01

**Authors:** Cristina Fante, Franziska Spritzler, Lori Calabrese, Nicole Laurent, Caroline Roberts, Sofia Deloudi

**Affiliations:** ^1^MetaboliCo GmbH, Bern, Switzerland; ^2^Keto-Check, Napa, CA, United States; ^3^Touchpoints180^™^, South Windsor, CT, United States; ^4^Family Renewal, Inc., Vancouver, WA, United States; ^5^Virta Health, Denver, CO, United States; ^6^Metabolic Health Consulting GmbH, Zurich, Switzerland

**Keywords:** β-hydroxybutyrate, ketone testing, ketogenic diet, nutritional ketosis, dietary adherence, personalized medicine, patient empowerment

## Abstract

Accurate assessment of dietary adherence and metabolic outcomes remains a critical challenge in most nutrition studies. Ketogenic metabolic therapies (KMTs) provide a unique advantage by inducing nutritional ketosis and enabling the use of ketone bodies as biomarkers of metabolic state. This narrative review investigates the role of ketone testing as an integral component of KMTs. We introduce the key biomarkers and testing modalities currently used and present a comprehensive overview of the use of capillary blood β-hydroxybutyrate (BHB) testing across diverse therapeutic areas. Capillary blood BHB testing plays a multifaceted role in KMTs: it enables objective monitoring of dietary adherence, supports the interpretation of clinical outcomes, and informs personalized treatment adjustments based on individual metabolic responses. Additionally, it may facilitate behavior change through real-time feedback. Broader implementation of ketone testing in both clinical and research settings will require thoughtful protocol design that accounts for individual preferences and tolerability, continued technological innovation to enhance user experience, and further research into the relationship between ketone levels and therapeutic outcomes.

## 1 Introduction

A major challenge in most nutrition intervention studies is the lack of objective biomarkers to accurately monitor dietary adherence and metabolic responses (1–3). Metabolomic profiling is emerging as an advanced method to detect the intake of specific foods, but its use is limited by inter-individual variability, the need for extensive validation, and poor translatability from research settings to routine clinical practice (2–4). Recovery biomarkers, such as doubly labeled water for energy intake and 24 h urinary nitrogen for protein intake, have been used as estimates of dietary intake, but their implementation is limited due to narrow applicability and logistical challenges (5, 6).

In the absence of reliable biomarkers, most studies of dietary patterns continue to rely on self-reported dietary intake data, which are inherently prone to recall bias, misreporting, and inter-individual variability (7–9). In intervention studies where outcomes depend on precise nutritional adherence, these limitations are particularly problematic.

**Figure 1:**
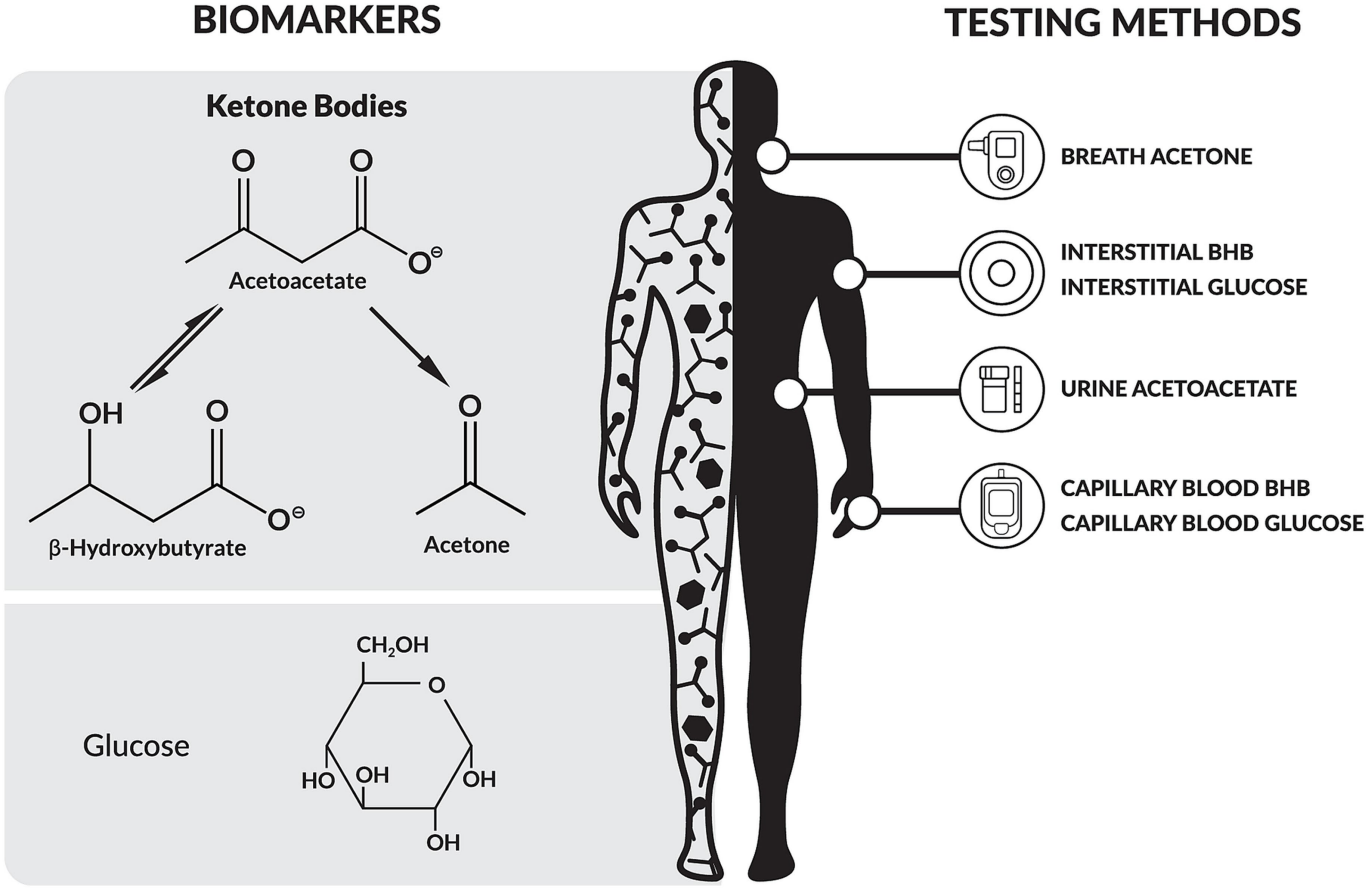
Overview of biomarkers and testing modalities relevant to ketogenic metabolic therapies (KMTs). BHB, β-hydroxybutyrate.

Ketogenic metabolic therapies (KMTs) provide a unique advantage in this regard. KMTs are dietary and lifestyle interventions designed to increase fat metabolism and ketone production, inducing the metabolic state of nutritional ketosis (10). The hallmark of nutritional ketosis is the elevation of the ketone bodies β-hydroxybutyrate (BHB), acetoacetate, and acetone, which can be objectively measured in blood, urine, and breath, respectively (Figure 1) (11). In particular, nutritional ketosis is defined by a precise BHB concentration range of 0.5–5.0 mmol/L in blood (12), thereby making BHB testing integral to the definition of KMTs.

Nutritional ketosis can be achieved with ketogenic diets that provide high fat, very low carbohydrate, and moderate to adequate protein intake. Therapeutic versions (such as the classic ketogenic diet, modified ketogenic diet, and modified Atkins diet) are used to manage epilepsy and other neurological conditions (13), each with distinct macronutrient ratios or carbohydrate restrictions (typically less than 30 grams of total carbohydrates daily). Ketogenic approaches are also applied to metabolic disorders like type 2 diabetes and obesity, typically delivering <10% of total energy from carbohydrates, which corresponds to approximately 30 to 50 grams of total carbohydrates daily (14). Variations include very low-calorie ketogenic diets (VLCKD) that are intended for short-term use (15), calorie-sufficient very low-carbohydrate diets that are suitable for long-term use (16), and intermittent fasting or time-restricted feeding protocols (17). Importantly, while extended fasting reliably induces ketosis, it may not be sustainable long term due to risks of lean mass loss and reduced resting metabolic rate (18). Even with their therapeutic potential, KMTs may lead to side effects such as gastrointestinal disturbances and electrolyte imbalances, and should therefore be implemented with appropriate clinical oversight.

In this narrative review we investigate the role of capillary blood BHB testing as an integral component of KMTs. First, we provide an overview of the key biomarkers relevant to KMTs, discussing their advantages, limitations and contexts of application (Section 2). Second, since monitoring of capillary blood BHB levels represents the current gold standard for assessing nutritional ketosis (11), we present a comprehensive review of its applications across different therapeutic areas, emphasizing testing modalities and impact on data interpretation (Section 3). Last, we discuss the role of capillary blood BHB testing in research and clinical practice in light of the reviewed literature, highlighting its potential to: (1) enable objective monitoring of dietary adherence; (2) support precise interpretation of clinical outcomes; (3) facilitate behavior change and empower patients; and (4) personalize treatment (Section 4).

## 2 Biomarkers and testing methods used in KMTs

Several biomarkers and testing methods are relevant to KMTs (Figure 1), each with specific advantages and limitations (Table 1).

**Table 1:** Comparison of currently available biomarker testing methods used in KMTs.

Biomarker	Biological sample	Advantages	Limitations	Ref.
Acetoacetate	Urine	Non-invasive, inexpensive, widely available, useful for initial adaptation phase.	Affected by hydration status. Become less reliable as ketosis continues.	(19–22)
Acetone	Breath	Non-invasive, portable, suitable for frequent monitoring.	Current metal oxide-based technology shows sensor stability and cross sensitivities with risk of false positives.	(23–27)
BHB (fingerstick)	Capillary blood	Accurate, reliable, reflects real-time metabolic state. Gold standard for monitoring of ketosis.	Invasive. Relatively costly (depending on testing frequency).	(28–32)
BHB (continuous)	Interstitial fluid	Indication of BHB trends and fluctuations.	Invasive. Relatively costly. Less accurate and less reliable than capillary ketone testing.	(33–35)
Glucose (fingerstick)	Capillary blood	Direct measure of glucose response to dietary and lifestyle interventions, relative low cost.	Invasive. Does not fully capture metabolic adaptation to ketosis.	(36)
Glucose (continuous)	Interstitial fluid	Indication of glucose trends and fluctuations.	Invasive. Less accurate and less reliable than blood glucose. Does not fully capture metabolic adaptation to ketosis.	(37–39)

Acetoacetate is a primary ketone body produced in the liver from fatty acid metabolism. It can be utilized directly for energy, enzymatically converted into BHB, or spontaneously decarboxylated into acetone. Acetoacetate is excreted in the urine. Urine dipsticks have been widely used for decades to detect elevated acetoacetate levels in diabetic ketoacidosis (DKA), largely due to their affordability and non-invasive nature. However, as individuals adapt to sustained ketosis, renal reabsorption of acetoacetate increases (19), reducing its urinary excretion and limiting the reliability of urine dipsticks for assessing ketosis (20). While capillary blood BHB testing and urine dipsticks have exhibited equal sensitivity for detecting DKA, BHB has been shown to have higher specificity (21). In the context of nutritional ketosis, the reliability of urine acetone measurements is further limited, as hydration status significantly affects accuracy (22).

Acetone is a volatile ketone body derived from the spontaneous breakdown of acetoacetate and is exhaled through breath, making it a non-invasive biomarker of ketosis (23). Studies have demonstrated that breath acetone is moderately correlated with plasma acetoacetate and plasma BHB, supporting its use as a reliable biomarker of ketosis (24). However, the accuracy of breath acetone measurements depends on multiple factors, including the performance of the breath analyzer [e.g., cross-sensitivities and poor stability of current metal oxide sensors (25)], the breathing maneuver used, and human variables such as breath volume, pattern, and temperature (26). Therefore, the clinical and research applications of acetone as a biomarker of ketosis remain limited by current technological constraints (27).

BHB is the most abundant ketone body in circulation, synthesized in the liver from acetoacetate through an enzymatic reduction (28, 29). BHB serves as a primary metabolic fuel during ketosis, supplying energy to the brain, heart, and muscles. Beyond its role as an energy substrate, BHB also functions as a signaling molecule, modulating inflammation, gene expression, and mitochondrial function (28, 29). BHB is measured in the blood and is considered the current gold standard for assessing ketosis (11). Historically, quantification relied on gas chromatography, mass spectrometry, and enzymatic assays, which provided high precision in clinical and research settings. Advances in technology have led to the development of modern capillary blood BHB meters, offering a convenient, rapid, and accurate alternative for home monitoring (30–32). As reviewed in the following section, capillary blood BHB testing is widely used in KMTs in both clinical and research settings.

Emerging technologies aim to expand BHB monitoring capabilities beyond point-in-time measurements. Continuous ketone monitors (CKMs) are wearable devices that measure BHB concentrations in interstitial fluid in real time. They operate via a subcutaneous sensor, which continuously samples interstitial fluid to assess BHB levels. CKMs offer continuous ketone data that may improve the detection and management of DKA, particularly in high-risk populations (33, 34). In people following KMTs, continuous ketone monitoring could provide feedback that may reveal circadian and behavioral patterns in ketone production not captured by conventional testing methods. However, challenges remain regarding sensor accuracy, calibration requirements, and the need for clinical validation before widespread adoption in both clinical and lifestyle contexts (33, 35). Currently, CKMs are available for consumer use only in selected markets.

In addition to ketone levels, glucose monitoring is frequently integrated into KMTs to evaluate glycemic regulation and metabolic adaptation. Beyond laboratory measurements, glucose can be self-monitored using capillary blood glucose meters or continuous glucose monitors (CGM), each with distinct implications and considerations.

Capillary blood glucose testing with a home glucose meter provides a practical and cost-effective approach for assessing glycemic responses to dietary and lifestyle modifications. This fingerstick-based method provides a single-point measurement of blood glucose levels. Traditionally, it has been extensively used in type 2 diabetes management and has been shown to improve clinical outcomes, particularly when combined with telehealth-based remote monitoring (36). However, similar to BHB testing, the perceived invasiveness of fingerstick measurements may reduce adherence to regular monitoring.

CGMs offer an alternative approach, measuring interstitial glucose levels in real time via a subcutaneous sensor, typically on the upper arm. These devices track glucose fluctuations and patterns continuously throughout the day and night, detecting episodes of hyperglycemia and hypoglycemia, and providing insights on individual responses to food intake, physical activity, and other lifestyle factors (37). Initially developed for diabetes management, CGMs are now increasingly popular among individuals without diabetes who wish to improve metabolic health and performance (38). However, CGM accuracy remains a concern, as studies indicate that CGMs may overestimate fasting and postprandial glucose levels compared to capillary blood glucose, with variability influenced by individual factors and the foods consumed (39).

While glucose monitoring alone (via fingerstick or CGM) may offer valuable metabolic insights, it does not capture the metabolic shift induced by KMTs or directly measure ketosis. The glucose ketone index (GKI) addresses this limitation by integrating both blood glucose and BHB levels into a comprehensive metabolic biomarker that reflects the balance between glycolytic and ketogenic metabolism. The GKI is calculated as the ratio of blood glucose level to BHB level (both in mmol/L) measured at the same time, with lower values indicating a greater metabolic shift toward fat oxidation and ketone utilization. Originally developed to monitor therapeutic ketosis in patients with glioblastoma (40, 41), the GKI has more recently been investigated as a tool for monitoring KMTs in mental health conditions such as depression and bipolar disorder (42, 43).

## 3 BHB testing in KMTs

Since monitoring of capillary blood BHB levels represents the current gold standard for assessing nutritional ketosis (11), we present a comprehensive review of its application in clinical research across a range of therapeutic areas, including diabetes, obesity, metabolic dysfunction-associated steatotic liver disease (MASLD), psychiatric conditions, neurological disorders, cancer, polycystic ovary syndrome (PCOS) and kidney disease. For each study, we highlight the testing protocol and, where available, report the original authors’ interpretation of the role of BHB data in informing adherence assessment, outcome interpretation, and therapeutic decision-making. An overview of the reviewed studies by therapeutic area is given in Table 2. While the increasingly compelling clinical outcomes associated with KMTs in these therapeutic areas are highly relevant, they have been reviewed extensively elsewhere (14, 44–47) and fall outside the scope of this review.

**Table 2:** Overview of reviewed studies using BHB testing in KMTs, by therapeutic area.

Therapeutic area	Study design (*n*)*	Frequency of BHB testing	Reported BHB range (mmol/L)	Adherence to BHB testing	Ref.
Type 2 diabetes (incl. prediabetes)	RCTs (2) Non-RCTs (1) Crossover (1) Pilot (1)	From daily to weekly	≥0.5	nr	(16, 53, 55–60)
Obesity	RCT (1) Pilot (1)	Occasional	≥0.3	nr	(62, 63)
Metabolic dysfunction-associated steatotic liver disease (MASLD)	Prospective (1)	Occasional	≥0.5	nr	(61)
Bipolar disorder	Pilot (2) Case report (1)	From daily to weekly	0.5–1.0	From <50 to 95%	(43, 66, 67, 72)
Depression	Case series (1) Case report (1)	Daily	1.1–3.2	79%	(68)
Schizophrenia	Pilot (1) Case report (1)	From daily to weekly	0.8–3.5	From <50% to ≥80%	(69, 72)
Obsessive compulsive disorder	Case report (1)	Daily	0.8	nr	(70)
Post-traumatic stress disorder	Pilot (1)	Daily (3 times)	≥0.5	98%	(71)
Autism spectrum disorder	Pilot (1) Prospective (1)	Occasional	0.8–2.2	nr	(73, 74)
Alzheimer’s disease	RCT (1) Crossover (1) Case report (1)	From daily to weekly	0.8–3.0	nr	(77, 80, 85)
Huntington’s disease	Case report (1)	Daily	0.9	nr	(78)
Amyotrophic lateral sclerosis	Case report (1)	Daily	0.77	nr	(79)
Parkinson’s disease	RCT (1) Pilot (1) Longitudinal (1) Feasibility (1)	From twice daily to weekly	0.5–2.0	nr	(81–84)
Epilepsy	RCTs (2) Prospective (2) Retrospective (3)	Occasional	2.0–6.0	nr	(90–96)
Cancer	RCT (1) Non-RCT (1) Prospective (1) Feasibility (2) Case series (1) Case reports (3)	From daily to occasional	0.3–6.3	nr	(40, 99–106)
Type 1 diabetes	Observational (1) Case Reports (2) Case Series (1)	From daily to multiple times a day	0.3–1.2	nr	(109–112)
Kidney disease	Pilot (1) Feasibility (1)	From daily to occasional	0.5–1.3	nr	(116, 117)
Polycystic ovary syndrome	Prospective (3) Retrospective (1)	From daily to weekly	0.5–1.7	nr	(119–122)
Ageing	Crossover (1)	Daily (3 times)	0.1–1.9	99%	(124)
Sports performance	Comparative (1) Crossover (1)	From daily to weekly	0.5–3.7	nr	(131, 132)

*(*n*) indicates the number of studies reviewed for each design type. RCT, randomized controlled trial; non-RCT, non-randomized controlled trial; nr, not reported.

### 3.1 BHB testing in KMTs for type 2 diabetes, obesity, and MASLD

KMTs directly target the core pathophysiology of insulin-resistant conditions, including type 2 diabetes, by shifting metabolism toward fat oxidation and ketone production (48, 49). Clinical studies have shown that KMTs can improve key metabolic markers such as glycated hemoglobin (HbA1c), fasting glucose, fasting insulin, and body weight, all of which contribute to enhanced insulin sensitivity (50). Additionally, ketone bodies, particularly BHB, exert anti-inflammatory effects offering further benefits in chronic metabolic diseases such as type 2 diabetes and obesity (51, 52). While the incidence of DKA in people with type 2 diabetes on KMTs is very low, at-home monitoring can help track BHB trends, especially in those who are at higher risk [e.g., individuals taking SGLT2 inhibitors (32)].

This section reviews the role of ketone monitoring in KMTs for type 2 diabetes, prediabetes, obesity, and MASLD, focusing on testing methodologies and their clinical and research implications. Studies have employed diverse testing protocols, including daily, multiple times per week, weekly, or periodic measurements.

#### 3.1.1 Frequent ketone monitoring (daily)

In a non-randomized study comparing a continuous remote care intervention for nutritional ketosis to standard diabetes care, individuals with type 2 diabetes who chose the ketogenic intervention were instructed to measure capillary blood BHB at home each day (53). A mobile app enabled real-time data transmission to the care team, with biomarker testing frequency adjusted over time based on individual needs. Mean lab-measured BHB at 70 days was 0.54 mmol/L. Within one year 96% of participants (*N* = 262) reported at least one home BHB reading ≥0.5 mmol/L (53). Within two years 61.5% of participants (*n* = 194) reported at least one BHB measurement ≥0.5 mmol/L (16). On average, BHB was ≥0.5 mmol/L for 32.8% of measurements over the two years. In sub-analyses of the two-year data, higher frequency of BHB ≥ 0.5 mmol/L was associated with larger increases in HDL-C, IDL II, and LDL I, and greater decreases in TG and mid-zone LDL particles (54). The authors stated that long-term tracking of BHB allowed them to investigate the relationship between the frequency of reported nutritional ketosis and the shift from LDL subclass phenotype B to phenotype A. In an extension study at five years, participants who maintained diabetes remission at year two tended to have higher mean BHB levels throughout the study compared to those who did not sustain remission (55).

#### 3.1.2 Mid-frequency ketone monitoring (weekly or multiple times per week)

In a three-month randomized controlled trial comparing a ketogenic diet to a moderate carbohydrate diet, participants in the ketogenic diet arm tested BHB at home twice a week with the goal of achieving detectable capillary blood ketones (56). By week 6, 75% of participants in the ketogenic diet group achieved a BHB level ≥0.5 mmol/L.

The Keto-Med crossover trial enrolled 40 participants with prediabetes or type 2 diabetes and assigned them to follow a ketogenic diet and a reduced carbohydrate Mediterranean diet for 12 weeks each in random order (57). During the ketogenic diet phase, participants used home capillary blood meters to measure fasting BHB three times per week and underwent fasting venous blood draws at seven time points for plasma BHB analysis. These two measures indicated whether participants restricted carbohydrate intake sufficiently to achieve ketosis and provided an objective marker of adherence to the diet. In a secondary analysis of the Keto-Med trial, researchers observed that during 85% of the study weeks, participants’ average BHB levels remained within the light nutritional ketosis range of 0.5–1.5 mmol/L (58).

Less frequent BHB monitoring has been used in smaller studies. In a pilot trial, 11 women recently diagnosed with type 2 diabetes followed a ketogenic diet for 90 days (59). Plasma BHB levels were measured weekly, with 10 out of 11 participants consistently meeting their weekly BHB targets and one participant meeting all targets by the second week. By week 12, the average BHB level was 1.3 mmol/L, reflecting sustained adherence to the diet.

In a six-month randomized controlled trial 58 individuals with type 2 diabetes followed a diet with less than 50 grams of non-fiber carbohydrates per day and measured capillary blood BHB at home two to three times per week to assess the effects of carbohydrate restriction on cardiovascular risk factors (60). At the end of the trial, no correlation was found between levels of ketosis and ≥5% increase in small, dense LDL particles.

#### 3.1.3 Occasional ketone monitoring (baseline and at study visits)

To investigate the effects of a ketogenic diet on hepatic steatosis, a 6-day trial was conducted in ten individuals with overweight or obesity (61). BHB levels were measured at baseline and on day 6, showing a tenfold increase from 0.1 mmol/L to 1.0 mmol/L. Increased fatty acid partitioning toward ketogenesis was linked to a higher hepatic mitochondrial redox state and reduced citrate synthase flux, indicating metabolic adaptations that may contribute to the reversal of MASLD through a ketogenic intervention.

Periodic BHB testing has also been used in the context of VLCKDs. In a pilot study involving 95 adults with obesity, the potential influence of sex on appetite responses to weight loss and ketosis was examined over an eight-week VLCKD intervention (62). BHB was measured at baseline, week 9, and week 13. Participants with undetectable BHB received targeted dietary counseling to improve adherence. By the end of the study, women had significantly higher BHB levels than men (1.174 vs. 0.783 mmol/L, *p* = 0.029), although no sex-based differences were observed in appetite-related hormone responses or subjective appetite ratings.

In another trial 45 individuals with obesity were randomized to a VLCKD and 44 to a standard low-calorie diet for four months (63). Capillary blood BHB was measured at baseline and at eight scheduled follow-up visits. BHB levels of at least 0.3 mmol/L were observed in 91.1% of participants in the VLCKD group, with peak levels reaching 1.15 ± 0.96 mmol/L at two weeks.

### 3.2 BHB testing in KMTs for psychiatric and neurodevelopmental disorders

The use of KMTs has been increasingly explored in the context of psychiatric disorders [i.e., bipolar disorder, schizophrenia, depression, and post-traumatic stress disorder (PTSD)] and neurodevelopmental disorders (i.e., autism spectrum disorder). Emerging research suggests that metabolic dysfunction, including glucose hypometabolism, mitochondrial impairments, and neuroinflammation, may contribute to the pathophysiology of these conditions (44, 64). Since ketone bodies serve as an alternative energy source for the brain, influence neurotransmitter balance, and modulate inflammatory pathways, KMTs have been hypothesized to provide therapeutic effects in these conditions (44, 64, 65).

This section reviews the role of ketone monitoring in KMTs for psychiatric and neurodevelopmental disorders, focusing on testing methodologies and their clinical and research implications. Studies have employed diverse testing protocols, including daily, multiple times per week, weekly, or periodic measurements.

#### 3.2.1 Frequent ketone monitoring (daily or multiple times per day)

A 6–8-week pilot study in individuals with bipolar disorder utilized daily BHB monitoring to assess adherence to a KMT and its metabolic effects (66). Of the 27 recruited participants, 20 completed the intervention. Ketone data collection during the intervention was 95% complete, demonstrating the feasibility of daily BHB testing in this population. These readings indicated that participants achieved light ketosis (0.5–1.0 mmol/L) within 1–7 days of starting the KMT and optimal ketosis (1–3 mmol/L) within 3–13 days. Participants maintained strong adherence to the therapy, as confirmed by 91% of available readings indicating at least light ketosis (≥0.5 mmol/L) and overall mean daily ketone levels of 1.3 mmol/L (median = 1.1 mmol/L).

The high-resolution dataset of ketone levels in this study enabled researchers to correlate outcomes with ketosis levels in a subset of 14 patients who provided reliable daily ecological momentary assessment data (43). Ketone levels were significantly (*p* < 0.001) positively correlated with mood (r = 0.21) and energy (r = 0.19) and inversely correlated with impulsivity (r = −0.30) and anxiety (r = −0.19), while no significant correlation was observed between ketone levels and speed of thought (r = −0.08).

A similar approach was used in recent case reports. A patient with bipolar disorder with treatment-resistant depressive symptoms followed a KMT and tested ketones daily (67). Testing compliance was high (89%) for the 21-week period and confirmed that nutritional ketosis was maintained at BHB values of around 1.0 mmol/L. A patient with chronic major depressive disorder showed 79% BHB testing compliance over 14 weeks, with blood BHB levels established at 1.1 mmol/L with highest recorded BHB level of 3.2 mmol/L (68). Two patients with schizoaffective disorder followed a KMT and tested ketones daily (69). Testing adherence was high in the first five weeks but declined in later weeks, contributing to an overall 63% testing compliance rate over 10 weeks. Nutritional ketosis was confirmed at ≥0.8 mmol/L with highest recorded BHB level of 3.5 mmol/L. Three patients with major depression and generalized anxiety disorder and complex comorbidities were treated with a personalized KMT and requested to monitor ketone levels and GKI daily (42). Ketosis was defined as BHB ≥ 0.8 mmol/L and GKI < 6. Biomarker testing demonstrated individual variability in metabolic adaptation, with some participants achieving stable ketosis rapidly, while others exhibited fluctuations before reaching consistent BHB levels. Interestingly, symptom improvements seemed to align with sustained ketosis, suggesting a potential relationship between metabolic state and clinical outcomes. Similarly, in a 12-week case study of KMT for obsessive-compulsive disorder and ulcerative colitis, daily testing confirmed sustained nutritional ketosis with average weekly BHB levels around 0.8 mmol/L and GKI values mostly ≤6 (70). Symptom improvements were associated with higher BHB levels, despite occasional dietary lapses and fluctuations in adherence.

A four-week study assessed the feasibility of a KMT in individuals with PTSD (*N* = 4), utilizing high-frequency capillary blood BHB monitoring to track adherence and individual metabolic response (71). Participants measured BHB three times daily. A day in ketosis was defined as mean daily BHB ≥ 0.5 mmol/L, and required adherence was defined as ≥75% of days in ketosis since ketosis was attained. Frequent biomarker testing verified dietary adherence, with 2% of measurements missing, and enabled personalized assessment of ketosis levels based on each patient’s metabolic response. Dietary composition impacted ketone levels, with one patient showing decreased BHB levels after low food intake or consumption of a commercial high-fiber bread.

#### 3.2.2 Mid-frequency ketone monitoring (weekly or multiple times per week)

A four-month pilot study assessed a KMT in bipolar disorder and schizophrenia (72). Weekly BHB testing objectively confirmed adherence, and allowed researchers to stratify participants based on metabolic response rather than self-reported intake. Of 23 participants, 14 were classified as fully adherent (≥80% of BHB readings ≥0.5 mmol/L), six as semi-adherent (60–80%), and one as non-adherent (<50%), demonstrating varying engagement with the diet. Findings suggested a dose–response relationship, with higher ketone levels associated with greater psychiatric improvements, though further research is needed.

#### 3.2.3 Occasional ketone monitoring (baseline and at study visits)

Occasional BHB testing was used in studies investigating the application of KMT in autism spectrum disorder. In a three-month open-label, observer-blinded clinical trial, blood BHB testing was performed at baseline and at 3 months (73). While all participants on KMT (*n* = 15) showed increased BHB levels, >50% demonstrated substantial to moderate improvement in behavior, and no correlation was observed between BHB levels and symptom changes. In a six-month pilot study of intermittent four-week KMT interrupted by two-week diet-free intervals, blood BHB testing was performed at the end of each KMT phase (74). During the ketogenic phases, BHB levels were maintained between 1.8 and 2.2 mmol/L, while in the diet-free phases, BHB declined to 0.8–1.5 mmol/L, demonstrating a metabolic change between dietary states.

### 3.3 BHB testing in KMTs for neurodegenerative disorders

Neurodegenerative conditions such as Parkinson’s disease, Alzheimer’s disease, mild cognitive impairment, Huntington’s disease, multiple sclerosis, and amyotrophic lateral sclerosis are characterized by progressive neuronal dysfunction, often linked to mitochondrial impairment, neuroinflammation, and metabolic dysregulation (75, 76). Emerging evidence suggests that KMTs may offer neuroprotective benefits by enhancing mitochondrial function, reducing oxidative stress, modulating inflammation, and providing an alternative energy substrate to neurons affected by glucose hypometabolism (45, 75).

This section reviews the role of ketone monitoring in KMTs for neurodegenerative conditions, focusing on testing methodologies and their clinical and research implications. Studies have employed diverse testing protocols, including daily, multiple times per week, weekly, or periodic measurements.

#### 3.3.1 Frequent ketone monitoring (daily)

Daily capillary blood BHB testing (consistently at bedtime) was used in a 12-week randomized crossover trial of a ketogenic diet in mild or early-stage Alzheimer’s disease (77). Of the 26 randomized patients, 21 (81%) completed the ketogenic intervention and 18 achieved sustained ketosis, demonstrating high adherence. While on the diet, patients achieved a mean BHB of 0.95 ± 0.34 mmol/L. The authors attributed the positive adherence rate partly to the use of ketone monitoring, which was easy to perform and allowed prompt recognition and correction of difficulties.

A similar approach was employed in two case studies in Huntington’s disease and amyotrophic lateral sclerosis. In a 48-week case study of a time-restricted ketogenic diet in Huntington’s disease (78), daily bedtime testing confirmed sustained ketosis with a mean BHB of 0.90 ± 0.57 mmol/L. Despite consistent dietary adherence, this patient’s ketone levels remained at the lower end of the target range, likely due to the use of exogenous insulin for his type 1 diabetes, which may have suppressed ketogenesis. Similarly, a patient with amyotrophic lateral sclerosis monitored BHB daily (at bedtime) during an 18-month dietary intervention with a 45-month follow-up (79). While the patient maintained a mean BHB of 0.77 ± 0.43 mmol/L, consistent with physiological ketosis, levels were at the lower end of the therapeutic range, which the authors state could potentially be due to hypermetabolism typical of the condition (which may have resulted in increased ketone utilization) or to the lean body composition of the patient (which may have limited fat availability for ketogenesis).

A daily ketone measuring routine (every morning, in a fasted state) provided essential confirmation of ketosis and supported appropriate dietary changes in a six-month case study of a patient with Alzheimer’s disease and Down syndrome undergoing KMT (80). Initially, after limiting carbohydrate intake to 75 g per day, the patient’s capillary BHB remained ≤0.2 mmol/L, indicating that nutritional ketosis was not achieved. In response, carbohydrate intake was further reduced to <20 g per day, leading to a consistent rise in serum ketones to 0.8–3.0 mmol/L. The caregiver noted that BHB testing was instrumental in confirming ketosis.

Daily BHB testing also proved feasible in studies of KMT in Parkinson’s disease. Metabolic assessment through bedtime BHB monitoring was implemented in an eight-week randomized controlled trial of a low-fat diet versus a ketogenic diet in Parkinson’s disease (81). The ketogenic diet group achieved a mean weekly BHB of 1.15 ± 0.59 mM, confirming consistent physiological ketosis, while the low-fat diet group exhibited negligible ketone levels.

Twice-daily plasma BHB measurements (fasting and postprandial) were used to assess metabolic adaptation to a ketogenic diet in the first week of a three-week randomized feasibility trial in Parkinson’s disease (82). BHB levels exceeded 0.5 mmol/L by day four, confirming early ketosis induction. However, subjects with metabolic syndrome or insulin resistance exhibited lower BHB levels, suggesting differences in the response to the intervention.

#### 3.3.2 Mid-frequency ketone monitoring (weekly or multiple times per week)

Weekly capillary blood BHB testing was implemented in a 12-week pilot study of a ketogenic diet in patients with Parkinson’s disease to monitor adherence (83). The mean BHB level over 12 weeks was 0.64 mmol/L. Interestingly, participants who maintained nutritional ketosis (>0.5 mmol/L) demonstrated greater improvements in symptoms of depression and anxiety compared to those who did not. These findings show how consistent ketone monitoring may offer valuable insights into metabolic adaptation and its potential relationship with symptom improvements. The authors highlighted weekly ketone testing as a study strength. Building on these findings, the same group conducted a 24-week longitudinal study in Parkinson’s disease (84), again performing weekly BHB testing to confirm dietary adherence. All participants submitted weekly ketone readings, indicating adherence and confirming acceptable levels of nutritional ketosis (BHB 0.5–2.0 mmol/L).

#### 3.3.3 Occasional ketone monitoring (baseline and at study visits)

In an 18-week pilot randomized crossover trial of a modified Mediterranean ketogenic diet versus an American Heart Association diet in Alzheimer’s disease (85), BHB was measured at key study time points and during diet education visits. At these time points, all participants on the ketogenic diet showed increased BHB levels, demonstrating a level of adherence to the intervention. Interestingly, individuals with mild cognitive impairment exhibited lower BHB levels than those with subjective memory complaints, despite similar dietary adherence (determined with daily food records). The authors hypothesized that patients with mild cognitive impairment may have higher ketone uptake into target tissues or reduced ketone production or may necessitate longer dietary exposure to achieve comparable BHB levels due to higher insulin resistance. Despite the low frequency, biomarker testing offered valuable insights into characterizing metabolic responses across cognitive impairment spectrums.

### 3.4 BHB testing in KMTs for epilepsy

Epilepsy is a neurological disorder characterized by recurrent, unprovoked seizures caused by abnormal electrical activity in the brain. Approximately one-third of individuals with epilepsy are considered refractory or resistant to the effects of antiseizure medications (86). The ketogenic diet has been used as an alternative therapy for drug-resistant epilepsy (DRE) since the 1920s (87). Its anticonvulsant effects are thought to involve increased GABA synthesis, enhanced neuroprotective signaling, and modulation of potassium, sodium, and calcium channels—mechanisms that collectively reduce neuronal excitability and oxidative stress (88). An increase in NAD^+^ in response to KMTs may also contribute to seizure reduction (89).

This section reviews the role of ketone monitoring in KMTs for epilepsy, focusing on testing methodologies and their clinical and research implications. Studies mostly employed periodic measurements.

#### 3.4.1 Occasional ketone monitoring (baseline and at study visits)

In a large retrospective study of 300 patients with drug-resistant epilepsy, BHB levels were assessed during fasting and throughout the first three months of a ketogenic diet (90). Patients underwent a fasting period of 12 to 48 h, with BHB monitored with high frequency (four times daily) during the first week, then at one and three months. BHB levels reached 2.0 mmol/L at 19 h, peaked at 4.2 mmol/L at 43 h, and stabilized by the three-month mark. Findings indicated that in cases where seizures persist, raising BHB to 4.0–6.0 mmol/L may be beneficial, while higher initial BHB levels may help patients needing rapid seizure control.

A 12-month non-blinded prospective study evaluated 18 children with DRE on a ketogenic diet, with serum BHB levels measured at 3, 6, and 12 months (91). A trend was observed between higher BHB levels and reduced seizure frequency, although statistical significance was not reached, likely due to the small sample size. However, the study concluded that BHB levels serve as a reliable indicator of dietary adherence.

In a prospective study, the serum metabolome of 14 children with DRE was analyzed before and after three months on a ketogenic diet (92). Serum BHB levels were measured at baseline and after three months, confirming a metabolic shift toward ketogenesis. After three months BHB levels reached 4.3 ± 1.9 mmol/L, consistent with sustained nutritional ketosis.

In a three-month retrospective study of 34 children with DRE on a ketogenic diet, serum BHB levels were measured at baseline and after 3 months (93). Post-treatment BHB levels were significantly elevated in both responders (4.70 ± 1.41 mmol/L vs. 0.55 ± 0.35 mmol/L, *p* < 0.001) and non-responders (2.00 ± 0.75 mmol/L vs. 0.58 ± 0.33 mmol/L, *p* < 0.001), with responders exhibiting significantly higher BHB levels than non-responders.

A two-month randomized controlled trial evaluated the effects of a modified Atkins diet (MAD) in 80 adults with DRE (94). Blood ketone levels were measured at one month and two months, with a BHB target of 2–4 mmol/L. By one month, 84% of patients achieved ketosis within a median of 4 to 4.5 days.

A randomized controlled trial enrolled 104 participants with refractory epilepsy and assigned them to one of two ketogenic diets: 51 to the classic ketogenic diet and 53 to the MAD for six months (95). Serum BHB levels were measured at baseline and at one, three, and six months. At three and six months, mean BHB concentrations in the ketogenic diet group were 3.74 mmol/L and 4.00 mmol/L, respectively, compared to 3.40 mmol/L and 3.70 mmol/L in the MAD group. While blood ketone levels were higher in the classic ketogenic diet group, the difference was not statistically significant.

In a retrospective study of 33 patients with refractory epilepsy on a ketogenic diet (96) BHB levels were measured during follow-up visits and correlated with seizure reduction at three and six months (*p* = 0.037 and *p* = 0.019). In contrast, urinary ketones from both clinic visits and daily home measurements showed no correlation with seizure reduction. These findings, also confirmed by previous studies (97), support the use of blood BHB as a more reliable indicator of therapeutic response to the ketogenic diet, even when measured infrequently.

### 3.5 BHB testing in KMTs for cancer

Cancer cells rely heavily on glycolysis for energy, even in the presence of oxygen—a phenomenon known as the Warburg effect (98). The ketogenic diet may target cancer’s metabolic vulnerability, particularly in glioma and other metabolically dysregulated cancers, by lowering glucose and insulin, reducing inflammation and oxidative stress, and enhancing tumor response to standard therapies (46).

This section reviews the role of ketone monitoring in KMTs for cancer, focusing on testing methodologies and their clinical and research implications. Studies have employed diverse testing protocols, including daily, multiple times per week, weekly, or periodic measurements.

#### 3.5.1 Frequent ketone monitoring (daily)

An 80-month case study documented a man with glioblastoma who followed a ketogenic diet (40). Written records of daily capillary blood ketone monitoring showed BHB levels typically between 1.0 and 6.0 mmol/L. After maintaining a GKI near or below 2.0 for over two years on a strict ketogenic diet, his GKI increased to 5 to10 with relaxed dietary adherence, coinciding with tumor progression. Implementing time-restricted fasting and a strict KMT regimen restored his GKI to 2.0 or below.

A similar case report documented the experience of a woman with glioblastoma who followed an intensive KMT program alongside standard treatment (99). The KMT regimen included prolonged fasting, time-restricted eating, and a modified ketogenic diet, with daily bedtime capillary blood ketone monitoring. In the first year, her average weekly BHB was 2.82 ± 1.43 mmol/L and average GKI was 1.65 (range: 0.52–5.97). In the second year, her average weekly BHB was maintained at 2.32 ± 0.67 mmol/L with average GKI of 2.02 (range: 1.16–5.38) and no disease progression noted on imaging. In the third year, average weekly BHB decreased to 1.64 ± 0.65 mmol/L and average GKI increased to 3.20 (range: 1.14–17.20) due to stress and reduced adherence to KMT, which coincided with tumor progression.

A case series reported the experience of 12 patients with cancers of the central nervous system who followed a ketogenic diet for 120 days (100). Among the eight patients who monitored BHB levels at home twice daily, most maintained ketone levels above 0.5 mmol/L throughout the study. The 30-day GKI ranged from 0.95 to 2.9, while the end-of-study GKI ranged from 1.7 to 5.3. The authors commented that the use of home capillary blood ketone monitoring and online data management tools facilitated accurate tracking of dietary adherence, providing strong internal validation and reducing concerns about self-reporting reliability common in dietary studies.

In a feasibility study, 20 women with stage IV breast cancer followed a six-month ketogenic intervention that included prepared meals and nutrition coaching, with daily home monitoring of BHB levels (101). Mean BHB levels were 0.8 mmol/L during the first three months and 0.7 mmol/L during the second three months, consistently within the target range of 0.5–4.0 mmol/L. All participants who completed the first three months achieved nutritional ketosis (BHB ≥ 0.5 mmol/L) and maintained it 90% of the time, despite the anti-ketogenic effects of chemotherapy and steroids, demonstrating strong dietary adherence.

A prospective study followed 18 patients with glioblastoma on a ketogenic diet for at least six months (102). Patients monitored their BHB levels pre-prandially in the morning and afternoon (daily during the first month and twice weekly thereafter) targeting BHB levels >3.5 mmol/L. In six detailed cases, most patients achieved nutritional ketosis within the first week and maintained BHB levels consistently above 2.0 mmol/L throughout the study.

#### 3.5.2 Mid-frequency ketone monitoring (weekly or multiple times per week)

Weekly BHB testing was explored in 10 patients who underwent KMT for advanced malignancies, including lung, breast, esophageal, colorectal, ovarian, and fallopian tube cancers (103). All patients achieved BHB levels >0.5 mmol/L, which was inversely correlated with insulin levels. The authors noted that the extent of ketosis, rather than calorie deficit or weight loss, was linked to stable disease or partial remission, while patients who achieved the lowest ketone levels experienced progressive disease.

A case study reported the experience of a woman with metastatic thymoma who followed a metabolic intervention consisting of periodic fasting combined with a modified ketogenic diet for two years (104). Capillary blood ketones were measured at home three times per week, with a mean two-year BHB level of 3.50 ± 1.27 mmol/L. Mean BHB levels during fasting periods were higher, measuring 6.31 ± 1.55 mmol/L. The authors noted that periodic fasting (lasting ≥ two days) appears to have a distinct therapeutic advantage by inducing more pronounced changes in ketone levels compared to calorie restriction or a ketogenic diet alone.

In a controlled, non-randomized trial, 18 patients with rectal cancer followed a ketogenic diet during radiotherapy, with the intervention length based on their individual treatment schedules (105). Capillary blood ketones were measured at least once weekly during radiotherapy. Median BHB levels peaked at 0.8 mmol/L in the first and fourth weeks of radiotherapy but declined over the following 2–3 weeks, with about half of the patients no longer reaching nutritional ketosis at the time of measurement.

#### 3.5.3 Occasional ketone monitoring (baseline and at study visits)

A 12-week randomized controlled trial evaluated the effects of KMT in 80 women with breast cancer, with 40 assigned to the ketogenic diet group and 40 to the control group (106). Serum BHB was measured during the first, third, and fifth chemotherapy sessions. At six weeks, 70.4% of KD participants had BHB levels >0.5 mmol/L, and 89% had levels >0.3 mmol/L. By 12 weeks, 66.7% maintained BHB levels >0.5 mmol/L, with 89% sustaining levels >0.3 mmol/L.

### 3.6 BHB testing in type 1 diabetes

Ketogenic and very low-carbohydrate diets are gaining attention in type 1 diabetes, an autoimmune disorder that destroys the insulin-producing beta cells, for their ability to improve glycemic control and reduce insulin requirements (107). However, careful ketone monitoring and clinical judgment are essential to distinguish nutritional ketosis from DKA, as the BHB ranges for these distinct metabolic states overlap. BHB values within the KMT target range (≥0.8 mM and ≥1.5 mM) have been associated with an increased risk of DKA in people not following KMT (108) or who are currently ill (33). With frequent testing and proper medical oversight, ketogenic and very low-carbohydrate diets may offer metabolic benefits in type 1 diabetes, though long-term safety and efficacy need further study.

This section reviews the role of ketone monitoring in KMTs for type 1 diabetes, focusing on testing methodologies and their clinical and research implications. Currently, ketone testing in people with type 1 diabetes has been published in case reports and short-term observational studies in which ketones were measured daily or multiple times per day.

#### 3.6.1 Frequent ketone testing (daily or multiple times a day)

A case study described a man with type 1 diabetes who maintained a ketogenic diet for 10 years (109). Over a 60-day monitoring period, his mean BHB level was 0.8 mmol/L (range: 0.3–1.5 mmol/L), remaining well below the diagnostic threshold for DKA, but within the range identified as associated with increased risk of DKA (108).

Another case study described a man newly diagnosed with type 1 diabetes who adopted a ketogenic diet (110). Over a three-day monitoring period, he recorded 18 BHB measurements ranging from 0.3 to 1.2 mmol/L. The authors noted that these ketone levels remained relatively stable throughout the day, with no concerning spikes indicative of metabolic maladaptation.

One case series examined the effects of combining fasting with exercise on ketone levels in eight adults, including two with type 1 diabetes (111). Participants trained for and completed a five-day, zero-calorie fast while walking or running for 100 miles. During the study, BHB levels ranged from 0.3 to 7.5 mmol/L and were similar between individuals with and without type 1 diabetes.

An observational study compared ketone levels in 15 people with type 1 diabetes following different habitual carbohydrate intakes, including six on very low-carbohydrate diets (<50 g/day) (112). Mean BHB levels in the very low-carb group were 1.2 mmol/L, well below the threshold DKA. However, a discrepancy between the reported mean (1.2 mmol/L) and the range (0.6–1.15 mmol/L) in this group warrants clarification.

### 3.7 BHB testing in KMTs for kidney disease

KMTs have been proposed as a valuable option for patients with chronic kidney disease (113, 114), in particular for those with autosomal dominant polycystic kidney disease (ADPKD). ADPKD is a hereditary, progressive kidney disorder characterized by the development of numerous fluid-filled cysts, that lead to organ enlargement, fibrosis, and gradual loss of kidney function (115).

Emerging research suggests that metabolic dysfunction plays a key role in the progression of ADPKD, with cyst cells exhibiting defective mitochondrial function, impaired fatty acid oxidation, and increased reliance on glucose metabolism (115). Given these characteristics, nutritional strategies that induce ketosis may offer a novel therapeutic approach by shifting cellular energy metabolism away from glycolysis and towards ketone utilization.

This section reviews the role of ketone monitoring in KMTs for ADPKD, focusing on testing methodologies and their clinical and research implications. Studies have employed both frequent and occasional testing protocols.

#### 3.7.1 Frequent ketone monitoring (daily)

A 16-week pilot study evaluated a KMT in 24 people with ADPKD using twice-daily capillary blood BHB testing to assess adherence and metabolic response (116). After an introduction phase, participants targeted therapeutic ketosis (BHB 1.5–3.0 mmol/L) in Phase 2 and low-level ketosis (BHB 0.5–1.0 mmol/L) in Phase 3 of the study. BHB testing confirmed that all participants reached ketosis (BHB ≥ 0.5 mmol/L) within days and maintained it throughout the study, with average BHB levels of 1.3 mmol/L in the first six weeks and 1.1 mmol/L in the final six weeks. BHB levels varied throughout the day and across participants, highlighting the dynamic nature of ketosis.

#### 3.7.2 Occasional ketone monitoring (baseline and at study visits)

In a three-month feasibility study (*N* = 66) of a KMT in ADPKD, 23 participants were randomized to receive a ketogenic diet and underwent blood BHB testing at three study visits (117). The primary combined endpoint was defined as a combination of adherence assessed by metabolic parameters (BHB ≥ 0.8 mmol/L for ketosis) and patient-reported feasibility. In total, 43% of participants met the criteria for feasibility, largely due to not consistently reaching the predefined BHB threshold of 0.8 mmol/L at all study visits. To refine adherence assessment, an alternative BHB threshold of ≥0.6 mmol/L on at least two of three visits was explored, increasing adherence classification to 78% of participants, with none of the control group reaching this target. Additionally, 91% of ketogenic diet participants maintained higher BHB levels than baseline at least twice during the study.

### 3.8 BHB testing in KMTs for PCOS

PCOS is a common and heterogeneous disorder, typically marked by hyperandrogenism, oligo-anovulation, and metabolic dysfunction, with a multifactorial pathogenesis often linked to insulin resistance, which contributes to ovarian dysfunction, inflammation, and metabolic complications (118). Growing evidence suggests that ketogenic diets may benefit individuals with PCOS by improving insulin sensitivity, reducing androgen levels, promoting weight loss, and restoring menstrual regularity, primarily by reducing hyperinsulinemia (47).

This section reviews the role of ketone monitoring in KMTs for PCOS, focusing on testing methodologies and their clinical and research implications. Studies have employed diverse testing protocols, including daily, multiple times per week, weekly, or periodic measurements.

#### 3.8.1 Frequent ketone monitoring (daily or multiple times per day)

In a pilot trial, 17 women with PCOS followed a VLCKD for 45 days and monitored daily capillary blood and urine ketones at home (119). Mean capillary blood ketone levels significantly increased from zero at baseline to 1.7 ± 0.58 mmol/L, showing adherence to the intervention.

In a double-blind prospective cohort study, 60 women followed ketogenic diet guidance for 12 weeks and monitored their BHB at home daily (120). Dietitians provided the participants with tailored advice based on serum ketone levels and changes in body weight.

#### 3.8.2 Mid-frequency ketone monitoring (weekly or multiple times per week)

In a pilot trial, 14 women with PCOS consumed a ketogenic Mediterranean diet for 12 weeks (121). Capillary blood ketones were measured every other day for the first six days and once a week thereafter. The mean BHB value was 1.77 ± 0.55 mmol/L from day 7 to day 84, showing adherence to the intervention.

In a retrospective study, 25 women with PCOS and obesity followed a very low-calorie ketogenic diet for 12 weeks (122). BHB levels were monitored weekly and maintained between 0.5 and 0.7 mmol/L.

### 3.9 BHB testing in ketogenic approaches for ageing and performance

Ketogenic approaches have gained interest for their potential to enhance longevity by promoting metabolic flexibility, mitochondrial efficiency, and reduced oxidative stress (123). As with the therapeutic application of KMTs, clinical studies on aging have used ketone monitoring to obtain objective verification of metabolic state.

For example, a non-randomized, open-label crossover trial (124) investigated the metabolic effects of nutritional ketosis in 10 healthy women, using daily BHB testing to assess adherence and metabolic fluctuations across three 21-day study phases. Participants underwent a six-month lead-in period with once-daily ketone measurements, followed by four daily testing time points during the study phases to capture metabolic responses throughout the day. Testing confirmed clear metabolic shifts across phases, with BHB levels significantly decreasing from 1.9 ± 0.7 mmol/L in the first ketosis phase to 0.1 ± 0.1 mmol/L in the ketosis suppression phase (*p* < 0.0001), then returning to baseline in the third study phase (1.9 ± 0.6 mmol/L). These results verified strict adherence to the dietary protocol. Despite variability in individual BHB responses, almost all readings during the first and third phases exceeded 0.5 mmol/L, the standard threshold for ketosis, while very few exceeded this range in the second phase. The study achieved 99.37% adherence in capillary BHB testing, with four participants completing all 252 required tests, demonstrating high feasibility of frequent BHB tracking in this population.

Ketogenic diets have been proposed as a strategy to enhance athletic endurance performance by promoting metabolic flexibility, optimizing fat burning, and reducing reliance on glycogen (125). While fat oxidation rates are typically measured as changes in respiratory exchange ratio, BHB testing has been used to monitor adherence to ketogenic interventions in athletes (126–128) and to assess the level of ketogenesis compared to control groups (129, 130). These measurements provided objective verification of metabolic state and ensured accurate evaluation of dietary effects on performance.

A recent crossover study (131) investigated the metabolic effects of a low-carbohydrate, high-fat diet on endurance performance in 10 triathletes for six weeks, using BHB testing to track ketosis and guide dietary adjustments. Testing on days 1, 3, 7, 14, 21, 28, 35, and 42 confirmed that all participants achieved nutritional ketosis (≥0.5 mmol/L) within one week, maintaining 0.6 ± 0.5 mmol/L by day 42. BHB levels were significantly higher in the low-carbohydrate, high-fat diet vs. high-carbohydrate diets (0.5 vs. 0.1 mmol/L; *p* < 0.001), remained elevated despite carbohydrate supplementation, and declined during exercise (−0.2 ± 0.1 mmol/L; *p* < 0.001), indicating ketone utilization. In this study, beyond monitoring adherence and guiding metabolic adjustments, BHB testing provided key insights into ketone availability, utilization, and contribution to energy metabolism during endurance exercise.

A six-day comparative study (132) investigated the metabolic effects of different ketogenic strategies on athletic performance (*N* = 25), using pre-breakfast BHB measurements to assess ketosis. Testing confirmed that BHB monoester supplementation elevated capillary blood BHB levels compared to carbohydrate intake at all post-consumption time points, validating its efficacy in raising circulating ketones. In contrast, a ketogenic diet stimulated endogenous ketogenesis, with fasted BHB levels rising from day 3 and peaking at 3.7 ± 0.8 mmol/L. These data show the utility of BHB testing in tracking metabolic adaptation and verifying ketosis in performance studies.

## 4 Discussion

Unlike most dietary interventions, which lack direct physiological biomarkers, KMTs induce a measurable metabolic state, nutritional ketosis, that is quantifiable through ketone body levels. Among these, BHB measured in blood (0.5–5.0 mmol/L) has emerged as the current gold standard biomarker (11, 12). Here, based on the literature reviewed above, we reflect on the role of BHB testing in both research and clinical practice. In particular, we critically discuss how BHB monitoring enables objective tracking of dietary adherence, supports interpretation of clinical outcomes, facilitates behavior change and patient empowerment, and contributes to the personalization of KMTs. We also consider the potential challenges of implementing BHB monitoring and propose areas for refinement and future investigation.

### 4.1 BHB testing to monitor adherence to KMTs

Clinical trials across a range of conditions consistently confirm the utility of capillary blood BHB testing as a reliable and objective biomarker of adherence to KMTs by providing direct physiological evidence of ketosis. BHB monitoring enables differentiation of metabolic responses across dietary interventions (81) and improves confidence in adherence reporting compared to self-reported dietary intake (72, 100).

Studies in type 2 diabetes (53), neurodegenerative conditions (77), psychiatric disorders (66, 72) and oncology (40, 99, 101) have reported that both BHB testing and KMTs are feasible and acceptable in the target populations.

The frequency and consistency of testing are critical for accurate adherence assessment. High-frequency testing, ranging from daily (43, 49) to multiple times per day (71, 124), provides the most precise and actionable feedback, confirming whether nutritional ketosis is achieved and maintained, and enabling timely dietary and lifestyle adjustments (53, 71).

However, daily testing may not be acceptable in all settings. In a pilot study of bipolar disorder, daily BHB monitoring confirmed strong adherence (66), yet some participants reported the protocol as burdensome (133). These findings highlight the need to balance data granularity with participant acceptability. Mid-frequency testing (e.g., two to three times per week) has proven feasible and effective in studies on obesity and mental health (58, 72). Occasional testing, typically at baseline and at study visits, provides valuable information but lacks the resolution to guide timely intervention (62, 73).

Importantly, studies may implement adaptive protocols, with testing frequency evolving over time. Several studies have adopted frequent testing in the initial phase to establish adherence, followed by reduced frequency as individuals become metabolically adapted or more confident in managing their diet and lifestyle (16, 55, 82, 90). Careful consideration should be given to the choice of testing frequency, ensuring it aligns with therapeutic goals and individual preferences, to minimize patient burden and maximize the long-term sustainability of KMTs.

### 4.2 BHB testing for the interpretation of metabolic responses and outcomes in KMTs

Beyond adherence monitoring, BHB testing offers insights into individual metabolic responses to KMTs. Several case studies and small clinical series have described how BHB levels vary in response to factors such as dietary composition (71), pharmacological treatments (78), and underlying disease state or physiological characteristics (85, 99). These observations have proven valuable for interpreting unexpected fluctuations in ketosis and adjusting dietary strategies to match individual needs.

Among larger studies, relatively few have directly investigated correlations between BHB levels and clinical outcomes. In type 2 diabetes, higher BHB levels have been associated with improved lipid profiles and a greater likelihood of sustained remission (16, 53, 55). In psychiatric conditions, preliminary evidence suggests a dose–response relationship, with higher BHB levels correlating with greater symptom improvement (42, 43, 72, 82). In oncology, case reports have observed that greater depth and consistency of ketosis were linked to disease stability or partial remission, whereas lower BHB levels were more often associated with continued disease progression (40, 99, 103).

An analysis of a continuous remote care program targeting nutritional ketosis identified a mean BHB level of approximately 0.5 mmol/L during the first 90 days of carbohydrate-restricted nutrition therapy as the optimal threshold for achieving ≥10% weight loss at 1 year, with higher average ketone levels associated with greater weight loss (134).

Although promising, these correlations remain exploratory and are often limited by small sample sizes and observational, retrospective designs. More robust, controlled studies are needed to determine whether specific BHB thresholds are necessary for or predictive of therapeutic benefit, and to what extent such relationships are generalizable.

### 4.3 BHB testing as a driver of behavior change, empowerment, and personalization

**Figure 2:**
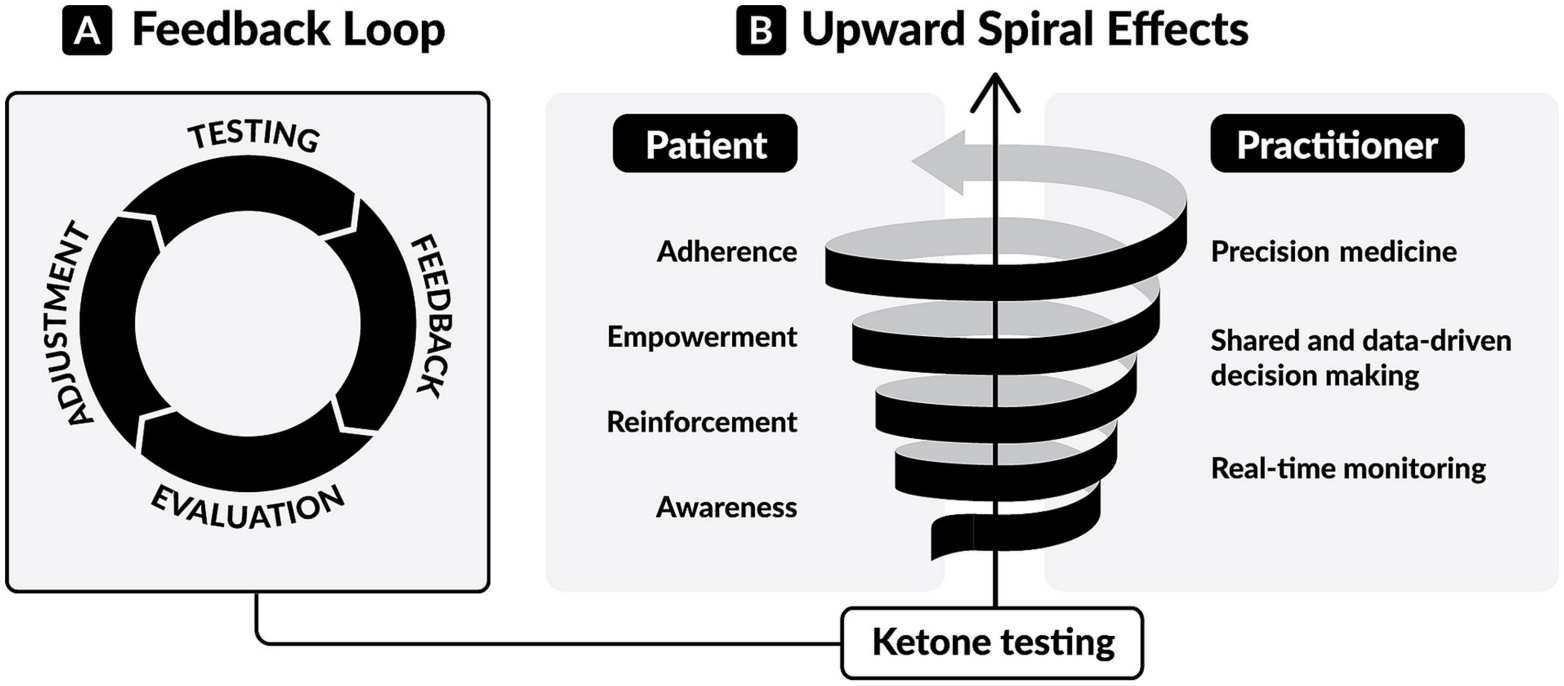
The dynamic of ketone testing in ketogenic metabolic therapies (KMTs). **(A)** The feedback loop of ketone testing. Individuals test, receive biological feedback, evaluate it, and adjust their dietary or lifestyle choices. Then they assess the resulting metabolic response by performing further testing. This real-time feedback provides actionable information for achieving and maintaining nutritional ketosis. **(B)** The upward spiral effects of ketone testing. For patients, consistent testing leads to awareness of personal metabolic responses, reinforcement of behaviors, personal empowerment, and adherence to KMTs. For healthcare professionals, consistent testing enables real-time, remote monitoring, shared decision-making based on personal data, and tailored interventions.

The utility of BHB testing extends beyond adherence tracking and outcomes interpretation. As a real-time biomarker, BHB may also play an active role in shaping behavior. At the core of BHB testing lies a simple yet powerful “feedback loop” (Figure 2): individuals test, assess the response, adjust their dietary or lifestyle choices, and test again to observe the resulting metabolic response. We speculate that this real-time feedback serves as a form of contingent reinforcement, where the measurable and immediate knowledge of BHB levels informs timely decisions, reinforces behaviors, and supports motivation and accountability, resulting in adherence to the KMT (135, 136). Over time, this develops into consistent reinforcement, fostering sustained behavioral change (137), enhancing engagement, and building a sense of empowerment through increased self-efficacy (138). This process drives an “upward spiral” (Figure 2) of positive behavioral and clinical change.

Research in behavioral science confirms that immediate biological feedback can strengthen motivation and facilitate sustained behavior change (135). Frequent and direct feedback has been associated with better outcomes in interventions targeting diet, exercise, and smoking cessation (139). Real-time feedback strategies, such as self-monitoring, have also been linked to greater engagement and adherence in digital health interventions (140). From a neuroscience perspective, biological feedback engages motivation and reward systems, particularly during the early phase of behavior change when motivation is high (136). Therefore, consistent BHB monitoring may serve as a neurobiologically aligned tool to support KMT adherence and long-term behavioral change through measurable reinforcement (135, 136, 140).

We also envision that, for healthcare professionals (HCPs), BHB monitoring enables a similar “upward spiral” (Figure 2) toward more precise and personalized care. Rather than relying on subjective reports, HCPs gain access to objective information that supports data-driven conversations, shared decision-making, and tailored interventions. When integrated with connected, cloud-based health platforms, BHB testing enables real-time, remote metabolic monitoring that precisely guides dietary adjustments, tracks therapeutic response (16, 53, 55), and permits detection of concerning clinical situations in high-risk individuals (32).

In summary, in our view, expanding the consistent and structured use of BHB testing may further enhance the promising clinical potential of KMTs by providing an objective assessment of adherence, improving the interpretability of outcomes, empowering individuals through actionable feedback, and enabling healthcare providers to deliver more personalized interventions.

### 4.4 Practical challenges and opportunities

While the reviewed evidence highlights the value of BHB testing in KMTs, its implementation in both research and real-world settings is not without challenges, warranting careful consideration, innovation, and ongoing refinement.

The invasive nature of capillary blood testing can act as a barrier, especially for those with needle phobia, fear of pain, or discomfort intolerance (141–143). Testing fatigue, particularly with daily monitoring, is well-documented in the diabetes literature (144), along with obsessive behaviors (145), and burdens associated with maintaining data integrity (133). Clinicians may address these psychological barriers using evidence-based strategies, such as cognitive behavioral therapy, gradual exposure methods, and distress tolerance interventions. Such approaches can effectively reduce anxiety, testing aversion, and obsessive behaviors associated with frequent biomarker monitoring (146–148). The use of CKMs may also offer relief from fingerstick testing burden while providing granular details about the metabolic milieu.

Although digital platforms and connected devices have improved data collection and sharing, technological limitations may still pose challenges, as some may find mobile apps and cloud-based monitoring tools cumbersome (116, 133), especially older adults or those with limited digital literacy.

Despite the increasing availability of capillary blood BHB meters and test strips, BHB testing remains relatively costly compared to other metabolic assessments such as blood glucose or urine ketone testing. This may limit accessibility, particularly in low-resource settings or for individuals requiring frequent testing.

As noted earlier, several important unknowns remain regarding BHB testing itself, including the optimal frequency of testing, the absence of standardized reference ranges across diverse populations, and the lack of defined targets for different therapeutic goals.

These challenges do not diminish the value of BHB monitoring but instead highlight the need for thoughtful design in both clinical protocols and real-world applications. Strategies such as reducing testing frequency after initial adaptation and providing structured onboarding, user training, and technical support can help mitigate these challenges and improve uptake, especially in underserved populations. Future technological improvements in non-invasive alternatives (e.g., breath acetone) may enhance accessibility and user comfort for those deterred by fingerstick testing. Research and innovation in both testing technologies and implementation strategies will be key to ensuring that BHB monitoring remains a feasible and empowering component of KMTs.

## 5 Methodological considerations and limitations of this work

This work presents a narrative review of the literature. We conducted a literature search in PubMed to identify interventional studies investigating the use of KMTs across a range of clinical areas (type 2 diabetes, obesity, MASLD, neurodegenerative diseases, psychiatric and neurodevelopmental conditions, cancer, epilepsy, type 1 diabetes, PCOS, kidney disease, aging, and physical performance). Studies were included if they incorporated capillary blood BHB testing as part of the methodology, independently of clinical outcomes. Studies involving KMTs without BHB testing were excluded. As such, we acknowledge the potential for unintentional bias in study selection, which is an inherent limitation of narrative reviews.

Although many of the included studies are clinical trials (Table 2), this review also includes some case reports and retrospective analyses with relatively small sample sizes. These study designs carry inherent limitations, including increased risk of bias and reduced generalizability. Nonetheless, they represent the current state of clinical research in the emerging field of KMTs.

Despite their limitations, these studies, when considered collectively, provide meaningful and consistent evidence of the role of BHB testing in the context of KMTs. As research in KMTs continues to grow, larger and more rigorously designed studies will be critical to further define and validate the role of BHB testing in both clinical and research contexts.

## 6 Conclusion

Capillary blood BHB testing provides an objective, quantifiable, and actionable measure of ketosis in KMTs. It offers real-time physiological feedback that reflects both dietary adherence and metabolic response. It supports behavior change, enables personalized care, and adds rigor to clinical research protocols. Evidence across diverse conditions confirms its value in both clinical practice and research settings, but further research is needed to investigate how BHB levels correlate to clinical outcomes across therapeutic areas. Considerate protocol design and continued innovation will be essential to integrate BHB monitoring more effectively into both clinical practice and research.
